# In Vitro and In Vivo Studies on the Structural Organization of Chs3 from *Saccharomyces cerevisiae*

**DOI:** 10.3390/ijms18040702

**Published:** 2017-03-25

**Authors:** Simon Gohlke, Subbaratnam Muthukrishnan, Hans Merzendorfer

**Affiliations:** 1 Department of Biology and Chemistry, University of Osnabrück, 49068 Osnabrück, Germany; simon.gohlke@rub.de; 2 Institute of Biology, University of Siegen, 57068 Siegen, Germany; 3 Department of Biochemistry & Molecular Biophysics, Kansas-State University, Manhattan 66506, KS, USA; smk@ksu.edu

**Keywords:** di-/oligomeric complexes, BiFC, chitin synthase, Chs3, proteinase K protection assays, topology, *Saccharomyces cerevisiae*, yeast

## Abstract

Chitin biosynthesis in yeast is accomplished by three chitin synthases (Chs) termed Chs1, Chs2 and Chs3, of which the latter accounts for most of the chitin deposited within the cell wall. While the overall structures of Chs1 and Chs2 are similar to those of other chitin synthases from fungi and arthropods, Chs3 lacks some of the C-terminal transmembrane helices raising questions regarding its structure and topology. To fill this gap of knowledge, we performed bioinformatic analyses and protease protection assays that revealed significant information about the catalytic domain, the chitin-translocating channel and the interfacial helices in between. In particular, we identified an amphipathic, crescent-shaped α-helix attached to the inner side of the membrane that presumably controls the channel entrance and a finger helix pushing the polymer into the channel. Evidence has accumulated in the past years that chitin synthases form oligomeric complexes, which may be necessary for the formation of chitin nanofibrils. However, the functional significance for living yeast cells has remained elusive. To test Chs3 oligomerization in vivo, we used bimolecular fluorescence complementation. We detected oligomeric complexes at the bud neck, the lateral plasma membrane, and in membranes of Golgi vesicles, and analyzed their transport route using various trafficking mutants.

## 1. Introduction

Chitin, a linear polymer of β-1,4-linked *N*-acetylglucosamine (GlcNAc), is a minor but important constituent of the yeast cell wall, which contributes to about 2% (*w*/*w*) of its dry weight [1]. During cell wall synthesis, chitin gets covalently linked to form higher-order complexes with β-1,3-glucan and β-1,6-glucan, which in turn are linked to mannans and cell wall proteins [2,3,4]. Chitin has vital functions during cell division and sporulation, particularly in the synthesis of the chitin ring at the bud neck, in primary septum formation and in spore wall formation, where chitin becomes deacetylated to form the outer chitosan layer [5]. Chitin synthesis in *Saccharomyces cerevisiae* is catalyzed by three membrane-integral family 2 glycosyltransferases (GT2) termed chitin synthase 1, 2 and 3 (Chs1, Chs2 and Chs3), of which the last enzyme accounts for the production of more than 90% of the total chitin [6].

Chs3 is a class IV fungal chitin synthase [7,8], and is regulated mainly at the posttranscriptional level [9]. It exhibits several posttranslational modifications, which are required for proper intracellular trafficking, such as ubiquitination and phosphorylation [10,11,12]. In the endoplasmic reticulum (ER), Chs3 is further palmitoylated by Pfa4, a modification which is necessary for its release from the ER [13]. ER exit is also dependent on Chs7, an ER chaperone which prevents Chs3 accumulation and aggregation in the ER [14]. Chs3 transport from the trans-Golgi network (TGN) to the plasma membrane (PM) involves the exomer complex, composed of the core protein Chs5 and Chs5-Arf1 binding proteins (ChAPs), which are believed to act as cargo receptors [15]. The ChAPs complex includes Chs6 and its three homologs Bch1, Bch2 and Bud7 [16,17]. Deletion of *chs5* or *chs6* leads to an accumulation of Chs3 in chitosomes, endocytic vesicles that are supposed to function as cell cycle-regulated Chs3 reservoirs [18].

The chitin synthase III complex (CSIII) consists of the catalytic subunit Chs3 and the regulatory subunit Chs4. It assembles even before chitin ring formation at the PM’s site of bud emergence. At the bud neck, CSIII interacts with the scaffold protein Bni4 that tethers Chs3 to the bud neck and anchors Chs3 to Cdc10 of the septin ring. However, *BNI4* deletion does not result in dramatic loss of CSIII activity [19]. As Bni4 is also a limiting determinant for recruiting the catalytic subunit of the type 1 serine/threonine protein phosphatase (Glc7) to the bud neck, Bni4-Glc7 complex formation appears to be required for subsequent targeting of CSIII [20]. Upon endocytosis, Chs3 is not degraded in the vacuole but enriched in chitosomes [9,21].

Chitin synthases are closely related to other membrane-integral GT2 enzymes such as hyaluronan synthases and cellulose synthases [22]. All these enzymes share several conserved motifs in the GT-domain: the Q(Q/R)XRW motif (X stands for any amino acid) which binds the terminal disaccharide acceptor of the glucan chain, and the (E/D)DX motif which is also essential for synthase activity [23]. A major breakthrough in analyzing GT2 enzymes was the crystallization of the BcsA-BcsB cellulose synthase complex from *Rhodobacter sphaeroides* revealing a narrow cellulose-conducting channel suggesting a model according to which chitin polymerization and translocation are tightly coupled processes [24]. This model may apply also for chitin synthases and the related rhizobial *N*-acetylglucosamine transferase NodC involved in the formation of lipochitin oligosaccharides [25,26]. In several algae and plants, cellulose synthases have been found to form complexes of different sizes and arrangements. Among them are hexameric rosette complexes, which were first reported in the 1980s [27,28]. They have been suggested to assist in fibrillogenesis, as up to multiple catalytic units simultaneously secrete sugar chains which spontaneously assembly into nanofibrils. Similarly, evidence has accumulated suggesting that chitin synthases can form oligomeric complexes. A yeast-two-hybrid (Y2H) study [29] reported homo-dimerization of Chs3 involving a dimerization domain that comprises the first 700 amino acids. In another Y2H study, the dimerization domain was narrowed down to the regions between amino acids 226 and 452 [30] and the N-terminal region comprising the first 171 amino acids [31]. Additional evidence that chitin synthases form oligomeric complexes were derived from biochemical studies in insects, that reported successful purification of active, oligomeric complexes of the midgut-specific chitin synthase 2 from *Manduca sexta* larvae (MsChs2) [32].

In this study, we performed bioinformatic analyses and protease protection assays to analyze the structure and topology of Chs3, which are still undetermined. We reveal significant information about the catalytic domain, the chitin-translocating channel and the interfacial helices in between. We further provide for the first time evidence for Chs3 di- or oligomerization in vivo using bimolecular fluorescence complementation assays (BiFC). We detected oligomeric complexes at the bud neck and the lateral plasma membrane, and in membranes of Golgi vesicles. BiFC analysis in strains defective in *chs7* and *pfa4*, two genes required for the ER exit of Chs3, revealed that Chs3 oligomerization may occur already at the ER. Finally, monitoring BiFC signals in *chs4*∆ strains, revealed that the regulatory Chs4 subunit of the chitin synthase III complex is not required for Chs3 trafficking and di-/oligomerization.

## 2. Results

### 2.1. Bioinformatic Analyses to Predict Membrane Topology and Three-Dimensional (3D) Structure Of Chs3 Reveals Gatekeeper and Finger Helices

**Figure 1:**
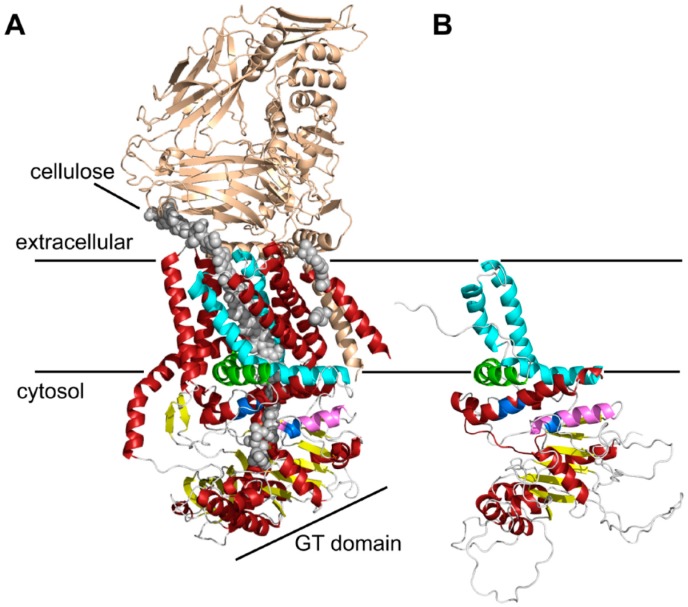
Three-dimensional (3D) structure predictions of chitin synthases generated by RaptorX. (**A**) Crystal structure of the BcsA and BcsB complex from *Rhodobacter sphaeroides* [24]. The BcsB protein is drawn in gold, the conserved motifs of the catalytic site (Q(Q/R)XRW, (E/D)DX; X stands for any amino acid)) are colored in blue, and the cellulose polymer is indicated by grey spheres; (**B**) RaptorX computed 3D structure of the C-terminal parts of Chs3. As a template for structure predictions the crystal structure of the bacterial cellulose synthase A (BcsA) was used. Note the highly conserved crescent-shaped, membrane-attached interfacial helix 3 (IF3) helix (green), which was originally predicted as a transmembrane helix (TMH), and the finger helix (pink) involved in polymer translocation. Conserved TMHs forming part of the polymer-conducting channel are colored cyan, other α-helices are depicted in red, and the BcsB subunit is shown in gold. Grey spheres illustrate the cellulose polymer.

Current knowledge on the mode of action of chitin biosynthesis, translocation and fibrillogenesis is limited due to the lack of structural data on chitin synthases. In a first approach, we examined membrane topology using various 2D structure prediction programs (Figure S1). The bioinformatic predictions revealed contradictory results with respect to the number of predicted transmembrane helices (TMHs), which ranged from 5 to 8, and the localization of soluble domains. Most programs used in this study predicted 2 TMHs at the N-terminal and 3–4 TMHs at the C-terminal region. For the central region, the number of predicted TMHs varied between 0 and 3. To obtain more accurate information about the topology of Chs3, a homology-based 3D structure prediction was carried out using RaptorX. The crystal structure of the BcsA-BcsB complex from *Rhodobacter sphaeroides* (Figure 1A; [24]) served as a template and allowed us to partially model the 3D structure of Chs3 based on sequence homologies in the C-terminal part (Figure S2).

This structure prediction largely resolved the catalytic domain of Chs3, which adopts a glycosyltransferase-A fold made of a mixed, seven-stranded β-sheet surrounded by seven α-helices (Figure 1B). It contains the highly conserved CHS signature amino acid sequences including QRRRW (product binding site), EDR (saccharide binding site) and WGDTR (required for in vivo activity), which all have been shown to be relevant for chitin synthesis by site-directed mutagenesis in yeast [33,34,35]. In addition, homology modeling disclosed two of the TMHs predicted at the C-terminal region. An additional homology-based structure prediction carried out with the protein homology/analogy recognition engine 2 (PHYRE2), led to similar results. However, the two C-terminal TMHs were not computed properly with the underlying algorithm (Figure S3). RaptorX delivered better results, as the spatial angle of these two helices resembled more the situation in BcsA (Figure 1B). Most notably, a third amphipathic α-helix in this region (green helix), which was expected to be a TMH as computed by all programs that we used for Chs3 topology predictions, does not span the membrane but is attached to the lipid bilayer from the cytosolic side. Strikingly, this crescent- shaped helix is highly conserved in GT2 enzymes (including BcsA from *R. sphaeroides*, where this helix is termed interfacial helix 3, IF3) and localized at the entrance to the transmembrane channel where it may act as a gatekeeper. An additional helix within the GT domain was identified carrying the EDR motif, which is homologous to the finger helix of BcsA. Notably, all these motifs were also identified in Chs2 (Figure S2). However, Chs3 lacks two THMs predicted for Chs2 between IF3 and the WGDTR motif.

### 2.2. Protease Protection Assays Suggest a Revised Model on Membrane Topology of Chs3

**Figure 2:**
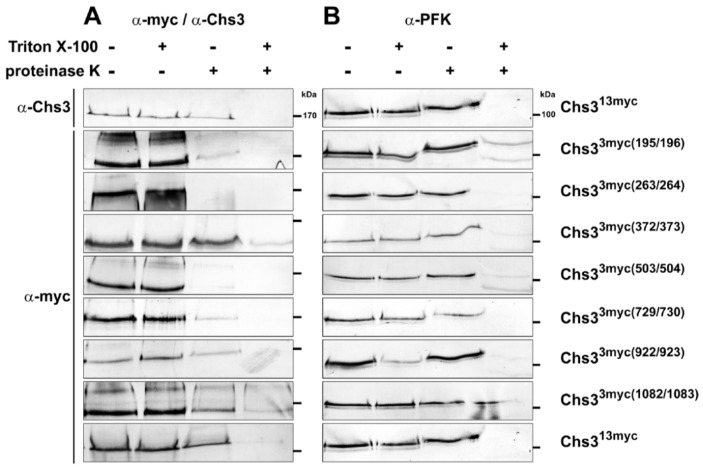
Topology determination of Chs3 using Proteinase K experiments with different myc-epitope tagged versions of Chs3. Cells expressing Chs3^13myc^ or different Chs3^3myc^ versions were converted into spheroplasts and treated with or without proteinase K in the presence or absence of Triton X-100. TCA precipitated proteins were solubilized and analyzed by sodium dodecyl sulfate polyacrylamide electrophoresis (SDS-PAGE) and Western blots using either: α-Chs3 or α-myc (**A**); and α-phosphofructokinase (PFK) (**B**) for immunostaining. Detection of PFK serves as a control for the integrity of the spheroplasts. Standard proteins are indicated with molecular masses given in kDa.

Due to the obvious limitations of computational algorithms in predicting topology, we conducted proteinase K protection assays to examine Chs3 membrane topology experimentally (Figure 2). For this purpose, Chs3 versions were generated, which contained either a 13myc-tag at the C-terminus or a 3myc-tag at strategically selected positions in regions that lie between adjacent transmembrane helical segments to probe whether these regions were located either on the intra- or extracellular side of the lipid bilayer. To examine whether the myc-tagged versions of Chs3 are functional in vivo, we conducted a Calcofluor White (CFW) drop test (Figure S4A). As shown, growth of the wildtype strain was completely inhibited by CFW, while the *chs3*∆ strain grew normally in the presence of CFW. In half of the cases, epitope tagging did not significantly impair in vivo function of Chs3. The *chs3*∆ strains expressing Chs3^13myc^, Chs3^3myc(503/504)^ or Chs3^3myc(729/730)^ grew like or almost like the wildtype. However, in those cells expressing Chs3^3myc(195/196)^ and Chs3^3myc(1082/1083)^, or Chs3^3myc(263/264)^ growth was far less (10- to 100-fold, respectively) reduced in comparison to the *chs3*∆ strain. Only in *chs3*∆ cells expressing either Chs3^3myc(372/373)^ or Chs3^3myc(922/923)^ growth was apparently not inhibited by CFW, suggesting that epitope-tagging compromised Chs3 functionality only in these two cases. To exclude that the observed variations in CFW sensitivity are due to differences in Chs3 expression, we performed immunoblots using α-myc and α-Chs3 antibodies, and α-phosphofructokinase (PFK) antibodies as a control (Figure S4B). The results indicate similar expression levels for all myc-tagged Chs3 versions (with somewhat lower expression for Chs3^13myc^ and Chs3^3myc(922/923)^ when reacted with α-Chs3 antibodies). The expression of the myc-tagged Chs3 versions was significantly higher than in the wildtype strain, because the transcription of the myc-constructs is driven by a strong PFK promotor. In order to address the question whether the supposed differences in chitin levels are actually real, we performed CFW stainings for wildtype and mutant cells (Figure S4C). We have shown previously that CFW fluorescence directly correlates with the chitin content determined chemically by the Elson–Morgan method [30]. With the exception of the *chs3*∆ strain, chitin is detectable in the wildtype and all created mutants, however, at different intensities, suggesting that chitin is synthesized and deposited by the epitope-tagged Chs3 versions in different amounts. The observed differences in fluorescence intensities are fully consistent with the results from the CFW sensitivity assays. The finding that chitin is still detectable in the cell walls of even those mutants which exhibit a very weak CFW sensitivity phenotype may indicate that Chs3 is transported to the plasma membrane but produces far less chitin. This assumption is supported by the results obtained from immunoblots shown in Figure 2. In those mutants exhibiting only a weak CFW sensitivity phenotype, proteinase K removes the outside-localized epitopes (e.g., Chs3^3myc(195/196)^) almost completely in the absence of Triton X-100; and when the epitope is localized at the inner side of the membrane (e.g., Chs3^3myc(372/373)^) the signal obtained in the absence of Triton X-100 has almost the same intensity as the controls in absence proteinase K. Together, these results suggest that most of the epitope-tagged Chs3 molecules are situated in the plasma membrane but produce chitin at different amounts.

Proteinase K treatments were performed using spheroplasts prepared from yeast cells expressing different versions of myc-tagged Chs3 in the presence or absence of membrane-permeabilizing Triton X-100. To test whether the used spheroplasts stayed intact during the preparation, we analyzed the possible degradation of the cytosolic phosphofructokinase (PFK) complex by immunostaining using α-PFK antibodies (Figure 2A, right panels). In all experiments the PFK-complexes (visible as double bands with only a small difference in molecular mass) were protected against proteinase K in non-permeabilized cells, indicating that the spheroplasts were fully intact and no unspecific protein degradation had occurred. Finally, we performed the proteinase K protection assays and detected the various epitope-tagged Chs3 versions in immunoblots from spheroplasts using α-myc antibodies. The controls that we performed in the absence of proteinase K (with or without Triton X-100) revealed no unspecific degradation of Chs3 and no significant Triton X-100 effects on band intensities (Figure 2A, left two lanes). In case of an extracellular localization of the tested myc-tag, we expected that Chs3 would be degraded irrespective of the presence or absence of membrane-permeabilizing Triton X-100. From the finding that Chs3 was degraded in *chs3*∆ cells expressing Chs3^3myc(195/196)^, Chs3^3myc(263/264)^, Chs3^3myc(503/504)^ and Chs3^3myc(729/730)^ in the presence or absence of detergent, we concluded that the corresponding regions of Chs3 at amino acid positions 191-341 and 476–893, that lie between adjacent transmembrane segments, are located extracellularly (Figure 2A, right two lanes). If the myc-epitope is localized at the intracellular site of the PM, Chs3 should be degraded only when the spheroplasts were treated with proteinase K in the presence of Triton X-100. These were exactly the results obtained when we analyzed *chs3*∆ cells expressing Chs3^3myc(372/373)^, Chs3^3myc(922/923)^, Chs3^3myc(1082/1083)^ and Chs3^13myc^ (Figure 2A, right two lanes). Similar results were gained when we immuno-detected Chs3^13myc^ with α-Chs3 antibodies directed against the first 171 N-terminal amino acids. Therefore, we conclude that the N- and the C-terminal regions (amino acid positions 1–167 and 1082–1165) are orientated towards the cytosol, as are the regions corresponding to amino acid positions 361–452 and 911–1014.

### 2.3. Bimolecular Fluorescence Complementation (BiFC) Experiments Demonstrate Di-/Oligomerization of Chs3 In Vivo

**Figure 3:**
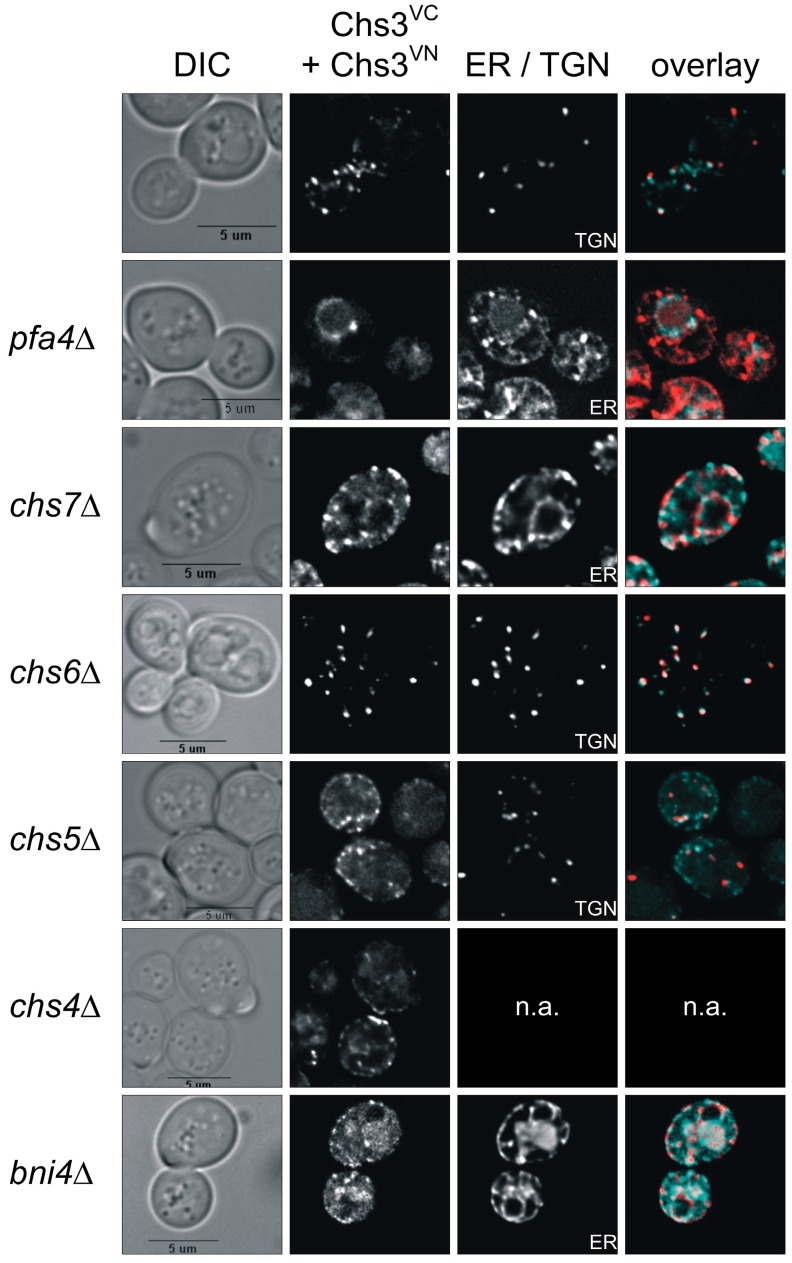
Visualization of Chs3 oligomerization in vivo using bimolecular fluorescence complementation (BiFC). Yeast strains with different genetic background expressing C-terminal tagged Chs3^VN^ and Chs3^VC^ were grown in selective media and synchronized by supplementing a logarithmically growing culture with α-factor for three hours. Cells were washed, transferred to fresh culture medium and analyzed after 90 min of incubation. Chc1^mCherry^ was used as a late-Golgi marker in *chs5*∆ and *chs6*∆ strains, and Sec66^mCherry^ as ER-Marker in all other strains. The markers were expressed from corresponding plasmids and under the control of their native promoter. BiFC signals are in cyan, and the mCherry-tagged markers are in red. *bni4*, *bud neck involved 4*; *chs3-7*, *chitin synthesis related*; DIC, differential interference contrast; ER, endoplasmic reticulum; *pfa4*, *protein fatty acyltransferase 4*; TGN, trans-Golgi network.

Previous studies performed in insect and fungal systems provided in vitro evidence that chitin synthases form di- or oligomeric complexes [29,30,31,32]. Based on these studies, we examined Chs3 oligomerization in vivo using BiFC analysis [36]. We used the N- and C-terminal halves of the Venus protein to reconstitute fluorescence upon Chs3 self-interaction. The N-terminal Venus part (VN) was genomically fused to the C-terminus of Chs3 (Chs3^VN^), while the C-terminal Venus part (VC) was fused to the C-terminus of Chs3 (Chs3^VC^) and expressed from the pRS415 plasmid under the control of the *MET25* promoter. The constructs/strains were tested for chitin synthase functionality and autofluorescence. CFW drop tests showed that Chs3 is functional because growth of the Chs3^VN^ strain as well as of *chs3*∆ cells expressing Chs3^VC^ was similar as in wildtype cells (Figure S5A). In order to exclude false-positive results in the subsequent BiFC experiments, Chs3^VN^ and Chs3^VC^ expressing cells were individually tested for autofluorescence. As shown in Figure S5B, no fluorescence signals could be detected at exposure times used in the subsequent BiFC experiments. When Chs3^VN^ and Chs3^VC^ were co-expressed in the same cell, BiFC signals became detectable indicating the formation of di- or oligomeric Chs3 complexes in vivo. The fluorescence was localized either at the bud neck, the PM or in the membranes of intracellular vesicles (Figure 3). In addition, some but not all of the vesicular signals, of which a few occurred close to the PM, co-localized with the late-Golgi marker Chc1^mCherry^ [37].

### 2.4. BiFC Experiments Using Truncated Chs3 Versions Reveal the Site of Self-Interaction

**Figure 4:**
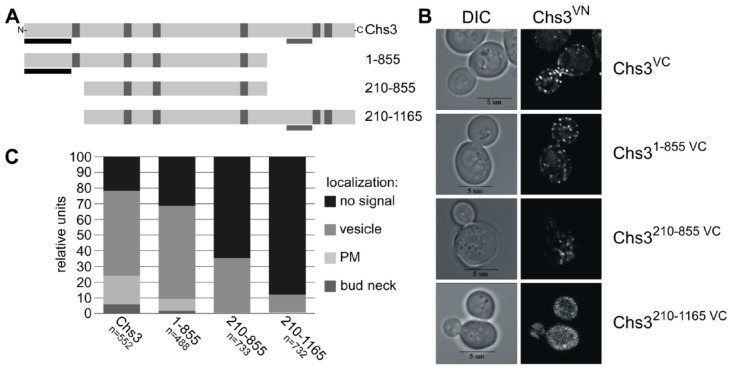
(**A**) Secondary structure of Chs3 and its truncated protein versions used for BiFC. Predicted TMHs are marked by vertical grey bars, known Y2H self-interaction sites by vertical black bars and the catalytic region by horizontal grey bars; (**B**) BiFC study to detect di-/oligomeric Chs3 by reconstitution the of fluorescence dependent on the interaction between genomic full-length Chs3 fused to the C-terminal half of Venus (VC) and different truncated Chs3 proteins fused to the N-terminal half of Venus (VN); (**C**) Quantitative determination of the intracellular localization of di-/oligomeric Chs3 determined by BiFC analysis.

Previous Y2H and crosslinking experiments with truncated Chs3 versions narrowed down the self-interaction site to an N-terminal region of Chs3 between amino acid positions 1–126 [31]. An additional self-interaction site was proposed based on the results from Y2H studies for the region between amino acid positions 226 and 452 [30]. Here, we performed BiFC experiments to determine which domains are responsible for the self-interaction in vivo. We co-expressed the genomically fused Chs3^VN^ together with various truncated Chs3^VC^ versions expressed from the corresponding pRS415 plasmid (Figure 4A). In contrast to previous interaction studies, we decided to use the full-length Chs3^VN^ protein as one of the two interaction partners to establish more native conditions. None of the truncated Chs3^VC^ fusion proteins displayed autofluorescence when expressed individually and inspected microscopically. Expression of the truncated Chs3^VC^ versions was confirmed by immunoblotting using α-GFP antibodies, which recognize the VC fusion protein (Figure S6B). Expression of full-length Chs3^VN^ was demonstrated before by CFW drop tests showing that the fusion product of Chs3 and VN has a functional chitin synthase (Figure S5A). In contrast, all truncated Chs3^VC^ versions lacked a functional chitin synthase in CFW drop tests (Figure S6C), partly because the catalytic domain was deleted and/or failed to form a chitin translocation channel, which is a prerequisite for chitin deposition in the cell wall and hence for CFW binding. Strikingly, co-expression of full-length Chs3^VN^ together with the different truncated Chs3^VC^ versions revealed BiFC signals in all cases, but at different subcellular localizations as determined in a quantitative analyses comprising hundreds of cells for each tested combination (Figure 4B,C). Evaluating co-expression of full-length Chs3^VN^ and the C-terminal truncated version Chs3^1–855 VC^ showed a reduction of BiFC signals at the bud neck and the PM when compared to cells co-expressing both full-length versions. Further shortening of the N-terminus (Chs3^210–855 VC^) resulted in a complete loss of BiFC signals at the bud neck and the PM, and a significant reduction of signals in the membranes of intracellular vesicles. Cells expressing a Chs3^VC^ version lacking only the N-terminus (Chs3^210–1165 VC^) showed BiFC signals only on the membranes of intracellular vesicles and this was observed in only about 10% of all evaluated cells. The results presented so far suggest that the interaction site may be located in the region of the N-terminus between amino acid positions 1 and 209, as previously proposed based on Y2H analyses and crosslinking experiments [38].

### 2.5. Tracking Chs3 Di-/Oligomerization in Strains Deficient in Chs3 Trafficking

Previous dithiobis(succinimidyl propionate)-cross-linking studies had suggested that Chs3 di-/oligomerization occurs already in the membranes of the Golgi and the ER [31]. Therefore, we addressed the question whether we would be able to track Chs3 di-/oligomerization in vivo along its trafficking route from the ER to the cell surface in a wildtype genetic background as well as in several deletion mutants that are defective in Chs3 trafficking. For this purpose, we co-expressed Chs3^VN^ and Chs3^VC^ in *chs7*∆ and *pfa4*∆ mutants, in which Chs3 fails to exit the ER, or in *chs5*∆ and *chs6*∆ mutants, in which sorting of Chs3 from trans-Golgi network vesicles to the cell surface is blocked (Figure 3). In contrast to the wildtype background, deletion of *pfa4* or *chs7* resulted in a complete absence of BiFC signals at the bud neck. Instead, BiFC signals were enriched in ER membranes as indicated by co-localization with the ER-marker Sec66^mCherry^ [39]. Image deconvolution revealed that the BiFC signals are found in clusters. This indicates that di- or oligomeric Chs3 complexes were formed already before sorting to the cell surface in special regions of the ER membrane. Deletion of *chs5* and *chs6*, both members of the exomer complex, led to a complete loss of BiFC signal at the bud neck and the PM when compared to the wildtype background. However, BiFC signals were detected as intracellular punctate structures co-localizing with the late-Golgi marker Chc1^mCherry^ in *chs6*∆ cells. Surprisingly, in *chs5*∆ cells only very few BiFC signals showed co-localization with the late-Golgi marker, whereas most of them reside in unidentified vesicles close to the PM. This finding strongly supports the central role of Chs5 in Chs3 tethering and maintaining integrity of the exomer complex. In our BiFC experiments with the deletion strain *chs4*∆ the distribution of BiFC signals resembled to that of cells with a wildtype background indicating that Chs4 does not affect the stability of di-/oligomeric Chs3 complexes nor is it required for Chs3 trafficking. Finally, we tested the influence of the scaffold protein Bni4 on Chs3 di-/oligomerization. We found, that in *bni4*∆ cells co-expressing Chs3^VN^ and Chs3^VC^, no distinct BiFC signals were formed. Instead, a more diffuse vacuolar signal became visible.

## 3. Discussion

**Figure 5:**
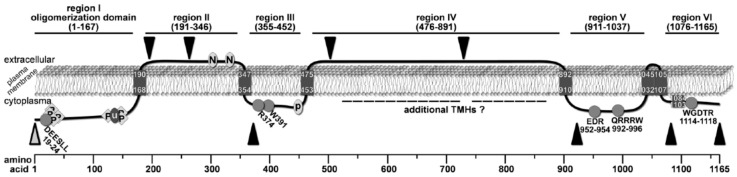
Revised topology model of Chs3 based on the combined results from previous interaction studies and our bioinformatic and biochemical analysis. C-myc insertions for proteinase K experiments are marked by black triangles, and the α-Chs3 antibody binding site is indicated by an open grey triangle. Conserved motifs are marked by grey circles or diamonds. Phosphorylation sites are indicated by P (diamonds), *N*-glycosylation sites by N (circle), the ubiquitination site by u (circle), and the palmitoylation site by P (circle).

As chitin is an increasingly important renewable raw material for the chemical/pharmaceutical industry, its biosynthesis is an economically relevant process. A detailed understanding of the molecular mechanism of chitin synthesis however can only be achieved when sufficient structural information is available. As no crystal structure for any chitin synthase has been reported to date, we still depend on genetic, biochemical and in silico data to draw conclusions on the mode of action of this enzyme. Genetic studies performed predominantly on Chs3 in the baker’s yeast have provided insight into the enzyme’s mode of action as well as into regulation of turnover and intracellular sorting. Although some of these studies have also investigated the topology of Chs3, many questions remained elusive, partially because of contradictory results, partially because of divergent interpretations, and partially because of insufficient consideration of data from literature. In this study, we performed homology modeling and protease protection assays, and combined our results with all available data from previous studies to arrive at an improved topology model of Chs3 (Figure 5).

By analyzing the accessibility of specific regions of this enzyme using proteinase K protection assays, we were able to map the topology of many soluble domains of Chs3. Based on these experiments, we demonstrated that the N-terminus (region I, aa pos. 1–167) faces the cytosol, a result which is in line with several findings from previous studies: (1) region I contains intracellularly localized DEESLL motif at aa positions 19–24 required for exomer-dependent transport [40]; (2) several phosphorylation sites have been reported within this N-terminal region by mass spectrometric analyses [8]; and (3) a large-scale study demonstrated that K136 is ubiquitinated and therefore a cytoplasmatic position was proposed for region I [12]. In contrast to many 2D topology prediction programs that computed two adjacent TMHs following region I, one program (Polyview/Sable) predicted only one TMH at position 168–190 with the consequence that region II (aa pos. 191-346) becomes orientated to the extracellular side of the plasma membrane. The extracellular localization of region II is supported by our proteinase K experiments using the mutants Chs3^3myc(195/196)^ and Chs3^3myc(263/264)^, as well as by two documented *N*-glycosylation sites (generally located extracellularly) at positions 303 and 332 [31,33,41]. Notably, our proteinase K assays using the mutant Chs3^3myc(372/373)^ suggests that this site is orientated towards the cytosol. Therefore, we postulate the presence of an additional TMH, which was not predicted by any of the 2D topology programs, in a region between the second extracellular *N*-glycosylation site at position 332 and the 3myc-tag at position 372. In contrast to a previously published topology model [31], this result, however, places region III (aa pos. 355-452) to the cytosolic site of the membrane.

Based on the assumption of an *N*-glycosylation site at position 371, it has been hypothesized that region III is exposed to the extracellular site [31]. However, the experimental data for this assumption are weak and do not really support that this putative *N*-glycosylation is utilized in vivo, particularly because the expected band shift between the N371Q mutant and wildtype Chs3 is not visible in the corresponding western blot after endoglycosidase treatment. The cytosolic orientation of region III is rather in line with the presence of intracellular sorting signals R374 and W391, which are essential for the transport of Chs3 to the PM through the alternative exocytic pathway [40]. Moreover, region III contains a putative palmitoylation site at position 446–451 [30], which may be used by Pfa4 to regulate the export of Chs3 from the ER [13]. Finally, Y2H studies suggested that the binding site of Chs3 for the cytosolic regulator protein Chs4, as well as for cytosolic domains of the zinc metalloprotease Ste24, resides within a region ranging from positions 226–452 [30]. As we have shown that the N-terminal half of this region belongs to the extracellular region II, we thus have narrowed down the interaction site for Chs4 and Ste24 to the cytosolic region III. Region III is terminated by a TMH at positions 453–475, which is predicted by several 2D topology programs and also postulated in the model by Sacristan et al. [31]. Proteinase K protection experiments with Chs3^3myc(503/504)^ and Chs3^3myc(729/730)^ revealed an extracellular orientation of region IV (aa positions 476–891). However, we cannot rule out the existence of additional TMHs in this regions, as high-throughput mass spectrometry experiments have identified phosphorylation sites at positions S537 and T538 [11]. Region V represents the catalytic domain, which is orientated towards the cytosol as also evidenced by the proteinase K experiment using the Chs3^3myc(922/923)^ mutant. This result is consistent also with the structural model derived from the crystal structure of the BcsA-BcsB complex. This arrangement requires the presence of a TMH between region IV and V, which may be located at position 892–910, but could be situated also at more N-terminal positions, as computed by some topology prediction programs.

Moreover, the results from our proteinase K assays using Chs3^3myc(1082/1083)^ and Chs3^13myc^ along with homology modeling revealed that the catalytic domain is followed by only two TMHs placing the C-terminal region VI to the cytosol. The most C-terminal helix, which was predicted to be a TMH by many topology programs, turned out not to span the membrane but forms a cytosolic interfacial α-helix (IF), which is amphipathic and crescent-shaped. Notably, this helix appears to be a general structural element controlling the entrance of the polysaccharide-conducting channel as deduced from the 3D structure of the BcsA-BcsB cellulose synthase from *R. sphaeroides*, where the homologous α-helix has been termed IF3 helix [24]. This helix is a constitutive part of the entrance of the cellulose-conducting channel formed by six TMHs and is also conserved in other GT2 enzymes. Homology-based 3D structure predictions of yeast chitin synthase Chs2 and the rhizobial *N*-acetylglucosamine transferase NodC as well as topology mapping using GFP and PhoA fusions of NodC demonstrated the existence of the IF3 helix in these GT2 enzymes [25]. In addition, investigations of the topology of the bacterial hyaluronan synthase from *Streptococcus pyogenes* revealed a homologous, non-membrane-spanning amphipathic α-helix at a similar position within the C-terminus [42].

The hypothesis that the IF3 helix is essential for chitin synthesis is strongly supported by experiments performed in the spider mite, *Tetranychus urticae*, in the diamond back moth, *Plutella xylostella*, and in the fruitfly, *Drosophila melanogaster*, which identified the resistance mutation that confers tolerance against the chitin synthesis inhibitors etoxazole, hexythiazox, clofentezine and diflubenzuron [43,44,45]. The mutation leads to a I–F (or I–M) substitutions in the IF3 helix, which presumably impairs binding of the inhibitor to the channel entrance site thereby preventing channel blockage by this compounds. Yeast cells are not susceptible to these class of channel-blocking chitin synthesis inhibitors (Figure S7), presumably because the homologous IF3 helix has slightly different characteristics and the corresponding mutation site (which is a leucine in yeast instead of an isoleucine) is shifted more into the center of the helix. However, this structural variation of the IF3 helix in Chs3 has a dramatic effect when we mimicked the resistance mutation by generating an L–F substitution at the corresponding position of the IF3 helix from Chs3. This mutation resulted in a complete loss of Chs3 functionality as indicated by the CFW resistance phenotype [43]. This finding emphasizes the important role of the IF3 helix at the channel entrance for chitin synthesis, and supports the hypothesis that chitin synthesis and chitin translocation are tightly coupled processes in vivo [26]. Another important interfacial helix in the BcsA-BcsB complex is the finger helix with an N-terminal TED motif that contacts the polymer’s terminal acceptor glucose [46]. The translocation of the cellulose takes place via a ratcheting mechanism in which the finger helix is moving up and down to push the polymer into the channel [46]. The finger helix was also computed in our 3D structure predictions for Chs3, where instead of the TED motif, chitin synthases possess the highly conserved EDR motif (Figure S2), which has been identified as a saccharide binding site [23]. The strong preservations and concordances found imply that the modes of catalysis and translocation are highly similar processes in chitin and cellulose synthases.

Several previous studies have reported di- and oligomerization for cellulose synthases from algae and plants, and partially cellulose synthases form hexagonal super-complexes known as rosette complexes [47]. First evidence for oligomerization of chitin synthases in insects has been provided by Maue et al. [32] by purifying an active, oligomeric chitin synthase complex from the midgut of *Manduca sexta*. Y2H and CoIP experiments performed in yeast also suggested that Chs3 is a self-interacting protein [29,30,31]. However, evidence that oligomerization really play a role in vivo was pending. In addition, the methods used for detecting the self-interactions have intrinsic limitations, such as the nuclear localization of the interaction partners in Y2H systems, or the fact that CoIP may also detect indirect physical interactions in larger protein complexes. Our BiFC results revealed for the first time di-/oligomerization and direct interaction of Chs3 in vivo. The oligomerization occurs at the bud neck, the TGN and other intracellular vesicles, probably within chitosomes. Depletion of the ER chaperone, Chs7, or the palmitoyltransferase Pfa4 does not impair oligomerization. Rather the localization and extent of interactions are affected. The oligomerization of Chs3 occurs already in the ER, which is in line with the results from CoIP experiments [31]. In comparison to the uniform distribution of Chs3^GFP^ in ER exit mutants [13], the BiFC signals for Chs3 appear in clusters on the ER membrane. This may indicate that only a subpopulation of Chs3 undergoes di-/oligomerization, and that this process depends on maturation and quality control.

Yeast strains with defects in the synthesis of Chs5 or Chs6, both components of the exomer complex involved in cargo sorting from the TGN to the PM, showed di-/oligomeric Chs3 complexes enriched in intracellular vesicles. In the *chs6*∆ strain, all BiFC signals co-localized with the fluorescent TGN marker Chc1^mcherry^, which is consistent with the immune-localization of Chs3^HA^ in *chs6*∆ strains [18], and suggests that Chs3 is transported already in an oligomeric form to the bud neck. Surprisingly, the result is different in *chs5*∆ stains, as only a subpopulation of Chs3 di-/oligomers co-localized with the TGN marker. Instead some punctate BiFC signals were located in unidentified vesicles close to the PM, which failed to reach the bud neck. An explanation might be that the depletion of Chs5 might have impaired TGN vesicle formation completely, as possibly homodimers of Chs5 form the organizational unit of the exomer complex, to which a varying number of Chs5 and Arf1 binding proteins (CHAPS, including Chs6) are bound [48]. As Chs3 cannot be packed into TGN vesicles in *chs5*∆ mutants, it might be recognized by alternate recruiting mechanisms such as the AP1-dependent or the poorly understood exocytic pathway [40]. If AP-1 is involved, it is likely that the BiFC signals represent early endosomes/chitosomes, and only a minor Chs3 fraction is retained within the TGN.

Surprisingly, the deletion of the regulatory subunit Chs4 had neither an influence on the oligomerization nor on the localization of Chs3 at the bud neck. Consequently, Chs4 is not necessary for targeting Chs3 to the bud neck, as already recognized by Reyes et al. [49], but also not required for oligomerization. Hence, how activation of Chs3 activity is facilitated by Chs4 remains elusive. Our BiFC experiments also underline the central role of the scaffold protein Bni4 for bud neck organization. As previously reported, Bni4 tethers Chs3 to the bud neck by interacting with Chs4 and septins in a Glc7-dependent manner [20,29]. This is why the loss of Bni4 results in diffuse intracellular signals of Chs3^mCitrine^ as demonstrated previously [50]. A similar diffuse distribution was observed in our BiFC experiments, suggesting delocalization of di-/oligomeric Chs3 complexes. To determine the oligomerization domain in vivo, we generated truncated Chs3^VC^ versions, with identical deletions used in previous in vitro experiments [31]. The quantification of BiFC signals in different cellular compartments revealed that the N-terminal region of Chs3 mediates di-/oligomerization. However, the function of Chs3 oligomerization is still puzzling. Our new topology model (see Figure 5) predicts that Chs3 has at least six TMHs, a number that is sufficient to form a chitin-translocating channel. Hence, oligomerization appears not to be required for chitin translocation. Rather, oligomeric chitin complexes may be involved in fibrillogenesis, as simultaneous secretion of nascent chitin chains in a pre-aligned manner may facilitate the formation of chitin nanofibrils.

## 4. Experimental Procedures

### 4.1. Chemicals

All chemical reagents were of analytical grade and purchased from local distributors. Sequencing was performed by Sequence Laboratories (Göttingen, Germany). Oligonucleotides (Table S1) were synthesized by Sigma-Aldrich (Steinheim, Germany). Calcofluor White (CFW), etoxazole, hexythiazox, clofentezine, diflubenzuron, α-factor and G418 were purchased from Sigma-Aldrich.

### 4.2. Media

Yeast extract peptone dextrose (YPD) medium was prepared with deionized water and contained 1% (*w*/*v*) yeast extract, 2% (*w*/*v*) peptone, and 2% (*w*/*v*) glucose. SD^−Ura^ medium and SD^−Leu^ medium contained 0,67% (*w*/*v*) yeast extract *w*/*o* amino acids, 2% (*w*/*v*) glucose and the corresponding drop out solution. Calcofluor plates were based either on YPD or yeast nitrogen base (YNB) medium containing additionally 0.2 mg/mL calcofluor (Sigma-Aldrich, Steinheim, Germany).

### 4.3. Construction of Plasmids and Strains

Oligonucleotides, plasmids and strains used in this study are listed in Tables S1–S3. Plasmid pRS415 VC was generated by integrating a polymerase chain reaction (PCR) amplicon, obtained from a reaction using the template pFA6a-VC-kanMX and the primers SG155 and SG156, into pRS415 Met25. Plasmids pRS415 Chs3^VC^, pRS415 Chs3^1-855 VC^, pRS415 Chs3^210-855 VC^, pRS415 Chs3^210-1165 VC^ were generated by integrating PCR amplicons, obtained from reactions using the template pRS415 Chs3 and the primers pairs SG106/SG161, SG106/SG160, SG159/SG160 and SG159/SG161, respectively, into pRS415 VC.

The plasmid pAG503 mCherry was generated by integrating of a PCR amplicon obtained from a reaction using the template pJJH71 ^mCherry^Chs4 and the primers SG219 and SG220, into pAG503. The plasmids pAG503 Sec66^mCherry^ and pAG503 Chc1^mCherry^ were generated by integrating PCR amplicons derived from genomic DNA using the primer pairs SG209/SG210 and SG236/SG237, respectively, into pAG503 mCherry.

Plasmid pJJH71 Chs3 was generated by the integration of a PCR amplicon derived from genomic DNA using the primer pair SG106/SG107 into pJJH71. Plasmid pJJH71 Chs3^13myc^ was generated by ligating a PCR amplicon obtained from a reaction using the template pFA6a-13myc-His3MX6 and the primer pair SG100/SG102 into pJJH71 Chs3. The plasmid pJJH71 Chs3^3myc(195/196)^ was generated by amplifying the vector pJJH71 Chs3 using phosphorylated primers SG221 and SG222 containing a *Sma*I restriction site. The 3myc tag was amplified using primers SG223 and SG224 containing *Sma*I restriction sites. The plasmid pJJH71 Chs3^3myc(263/264)^ was prepared by amplifying the vector pJJH71 Chs3 using phosphorylated primers SG238 and SG242 containing *Sma*I and *Sac*I restriction sites, respectively. The 3myc tag was amplified using primers SG232 and SG244 containing *Sma*I and *Sac*I restriction sites. Plasmid pJJH71 Chs3^3myc(372/373)^ was generated by integrating first the 3myc amplicon, obtained from a reaction using the template pJJH71 Chs3^13myc^ and the primers SG141 and SG142 (containing *Xba*I and *Sma*I restriction sites, respectively), into pJJH71 Chs3^13myc^. Next, the C-terminal part of Chs3 was integrated, obtained from a reaction using the template pJJH71 Chs3^13myc^ and the primers SG143 and SG144 containing *Sma*I and *Sph*I restriction sites, respectively. The plasmid pJJH71 Chs3^3myc(503/504)^ was prepared by amplifying the vector pJJH71 Chs3 using phosphorylated the primers SG234 and SG235 both containing a *Sma*I restriction site. The 3myc tag was amplified using primers SG232 and SG233 containing a *Sma*I restriction site. The plasmid pJJH71 Chs3^3myc(729/730)^ was prepared by amplifying the vector pJJH71 Chs3 using phosphorylated primers SG240 and SG243 containing SmaI and SacI restriction sites, respectively. The 3myc tag was amplified using primers SG232 and SG244 containing *Sma*I and *Sac*I restriction sites as well. The plasmid pJJH71 Chs3^3myc(922/923)^ was generated by insertion of the 3myc tag into the CDS of Chs3 using the native *Acc65*I restriction site. The 3myc tag was amplified using primers SG215 and SG216 containing an *Acc65*I restriction site as well. The plasmid pJJH71 Chs3^3myc(1082/1083)^ was prepared by amplifying the vector pJJH71 Chs3 using phosphorylated primers SG225 and SG226 both containing a *Sma*I restriction site. The 3myc tag was amplified using primers SG232 and SG233 both containing a *Sma*I restriction site.

Standard techniques were used for cultivation and genetic manipulation of yeast cells. Yeast transformation was done as described [51]. All PCR-based chromosomal operations were confirmed by colony PCR. *S. cerevisiae* wildtype (BY4741) and the isogenic knock-out strain *chs3*Δ were obtained from the EUROSCARF strain collection (Frankfurt, Germany). The strain BY4741 *CHS3^VN^* was generated by integration of a PCR amplicon derived from pFA6a-VN-His3MX6 generated with primer pairs SG132 and SG146. The deletion strains BY4741 *CHS3^VN^ CHS5*Δ, BY4741 *CHS3^VN^ CHS6*Δ, BY4741 *CHS3^VN^ CHS7*Δ, BY4741 *CHS3^VN^ BNI4*Δ, and BY4741 *CHS3^VN^ PFA4*Δ were generated by complete ORF deletion using the KlURA3 deletion cassette amplified from the deletion marker plasmid pUG72 (for detail see Supplemental Table S2). BY4742 *CHS3^VN^ CHS4*Δ was generated by mating BY4741 *CHS3^VN^* and BY4742 *CHS4*Δ. Resulting diploid strains were sporulated, and segregants carrying double mutations were selected on SD-His plates containing 200 µg/mL G418.

### 4.4. Complementation Assays in Yeast

The functional analysis of the Chs3 fusion proteins was carried out by a CFW drop test. For this purpose, *chs3*Δ cells expressing the respective Chs3 fusion protein were cultured overnight and plated in serial dilutions onto CFW/SD-Leu plates. The wildtype and the *chs3*∆ strains, both containing the empty vector, served as controls. Growth of the strains was monitored after 24 h of incubation at 30 °C.

### 4.5. α-Factor Synchronization

Yeast α-factor was used to arrest MATa cells at the G1/S-phase boundary by inhibiting Cln-Cdc28 activity, and synchronization was performed as published [52]. Yeast cultures were grown to early log phase and then α-factor (Sigma-Aldrich, Steinheim, Germany) was added to a final concentration of 2.5 μg/mL. Cells were incubated at 30 °C for 3 h to arrest cells in the G1 phase. To release the cells from the arrested state, the α-factor was removed by centrifugation of the culture (3 min, 1500× *g*), and removal of the supernatant. The cell pellet was washed twice with distilled water and finally resuspended in fresh culture medium. After growth at 30 °C, the cells were analyzed at different time points under the microscope.

### 4.6. Image Acquisition

Fluorescence microscopic images were obtained with an IX70 microscope (Olympus, Hamburg, Germany) using Chroma fluorescence filters, an Olympus ×100 UPlanApo (NA 1.35) and a CoolSNAP HQ2 camera (Roper Scientific, Tucson, AZ, USA). Images were captured using MetaMorph 6.2 software (Molecular Devices, Toronto, ON, Canada) and Huygens Essential (Image Solutions, Preston, UK) version 3.7 was used for deconvolution.

### 4.7. Proteinase K Protection Assays

Yeast cells expressing myc-tagged Chs3 versions were grown overnight at 30 °C in SD-Ura media until the early logarithmic phase was reached. Spheroplasts were generated by treating the cells with 2.5 µg/µL Zymolyase 100T (MP Biochemicals, Eschwege, Germany) for 60 min at 37 °C in spheroplast buffer (1 M Sorbitol, 7.5 mM DTT, pH 6.8). Spheroplasts were centrifuged (600× *g*, 1 min at 4 °C) and washed three times with ice-cold Sorbitol (1 M). For proteinase K digestion, the spheroplasts were transferred to four reaction tubes (2 mg wet weight/tube), resuspended in 500 µL ice-cold sorbitol (1 M), and treated with or without proteinase K (1.5 mg/tube) (Roche, Mannheim, Germany) in the presence or absence of 0.4% (*v*/*v*) Triton X-100. After 5 min on ice the reaction was stopped by the addition of 12.5% (*v*/*v*) ice-cold TCA and stored at −80 °C. The samples were thawed on ice, centrifuged (17,900× *g*, 10 min, 4 °C) and washed twice with ice-cold 80% acetone (*v*/*v*). The pellets were resuspended in 1% (*m*/*v*) SDS/NaOH (0.1 M), Laemmli buffer was added [53], and the samples were boiled for 3 min at 98 °C.

### 4.8. Sodium Dodecyl Sulfate Polycarylamide Electrophoresis (SDS-PAGE)/Western Blot

SDS-polyacrylamide gel electrophoresis and semidry electroblotting onto nitrocellulose membranes were performed as described by [30]. Blotted proteins were incubated with α-myc (1:100; AbD Serotec, Düsseldorf, Germany), α-PFK (1:10,000; kindly provided by Jürgen Heinisch, Osnabrück, Germany), α-Chs3 antibodies ([15], kindly provided by Anne Spang, 1:1000), or α-GFP antibodies (1:1000; Roche). As secondary antibodies α-mouse or α-rabbit antibodies conjugated to alkaline phosphatase were used (1:30,000; Sigma-Aldrich).

### 4.9. Bioinformatics

Predictions of Chs3 topology were done with TMpred [54], Polyview [55], Phobius [56], DAS TM filter [57], HMMTOP [58], TMHMM 2.0 [59], TMHMM 1.0 [60], and Philius [61]. Homology-based 3D structure predictions were made with PHYRE2 [62] and RaptorX [63]. Visualization of 3D structural models was performed with PyMOLv1.3.

## 5. Conclusions

Although Chs3 has been extensively investigated for decades, its mode of action is largely unknown mainly due to the lack of structural data obtained from crystallographic analyses. In silico analyses and protease protection assays performed in this study revealed significant information about the catalytic domain, the chitin-translocating channel and the interfacial helices in between. In addition, we could clearly demonstrate that a crescent-shaped, amphipathic α-helix at the C-terminus of Chs3, which is predicted to be a transmembrane helix by topology prediction programs, does not span the membrane but is attached to its cytosolic side. As computational prediction of enzyme topology is significantly limited, biochemical assays as performed in our study are still required in order to address questions regarding structure and topology of membrane bound enzymes. Using BiFC, we further show that Chs3 forms di- or oligomeric complexes in vivo. We detected oligomeric complexes at the bud neck and the lateral plasma membrane, and in membranes of Golgi vesicles. The fact that these results recapitulate some earlier findings obtained from in vitro studies on Chs3 di/-oligomerization underlines that BiFC can be a reliable method to analyze protein interactions in living cells. Using this method in different genetic backgrounds largely extents its capability.

## Supplementary Materials

Supplementary materials can be found at http://www.mdpi.com/1422-0067/18/4/702/s1.
