# Supplementary material for: In Vitro and In Vivo Studies on the Structural Organization of Chs3 from Saccharomyces cerevisiae
Source: Int J Mol Sci. 2017 Mar 25;18(4):702. doi: 10.3390/ijms18040702 (PMC5412288; doi:10.3390/ijms18040702)
Supplement: Supplementary file 1 [file ijms-18-00702-s001.zip › Supplemental Table 3.docx]

**Supplemental Table 3.** Plasmids

| **Name** | **source** |
| --- | --- |
| **ORF deletion** | |
| pUG72 | Euroscarf [1] |
| **BiFC experiments** | |
| pFA6a-VC-kanMX | Euroscarf [2] |
| pFA6a-VN-His3MX6 | Euroscarf [2] |
| pRS415 Met25 | Laboratory collection |
| pRS415 VC | This work |
| pRS415 Chs3^VC^ | This work |
| pRS415 Chs3^VC 1-855^ | This work |
| pRS415 Chs3^VC 210-855^ | This work |
| pRS415 Chs3^VC 210-1165^ | This work |
| **Fluorescence organelle marker construction** | |
| pJJH71 ^mCherry^Chs4 | Laboratory collection |
| pAG503 | Laboratory collection |
| pAG503 mCherry | This work |
| pAG503 Mnn9^mCherry^ | This work |
| pAG503 Sec66^mCherry^ | This work |
| **Proteinase K experiments** | |
| pJJH71 | J. Heinisch [3] |
| pFA6a-13myc-His3MX6 | Euroscarf [4] |
| pJJH71 Chs3 | This work |
| pJJH71 Chs3^13myc^ | This work |
| pJJH71 Chs3^3myc(195/196)^ | This work |
| pJJH71 Chs3^3myc(263/264)^ | This work |
| pJJH71 Chs3^3myc(373/373)^ | This work |
| pJJH71 Chs3^3myc(503/504)^ | This work |
| pJJH71 Chs3^3myc(729/730)^ | This work |
| pJJH71 Chs3^3myc(922/923)^ | This work |
| pJJH71 Chs3^3myc(1082/1083)^ | This work |

1. Gueldener, U.; Heinisch, J.; Koehler, G.J.; Voss, D.; Hegemann, J.H., A second set of loxp marker cassettes for cre-mediated multiple gene knockouts in budding yeast. *Nucleic acids research* **2002**, *30*, e23.

2. Sung, M.K.; Huh, W.K., Bimolecular fluorescence complementation analysis system for in vivo detection of protein-protein interaction in saccharomyces cerevisiae. *Yeast* **2007**, *24*, 767-775.

3. Raben, N.; Exelbert, R.; Spiegel, R.; Sherman, J.B.; Nakajima, H.; Plotz, P.; Heinisch, J., Functional expression of human mutant phosphofructokinase in yeast: Genetic defects in french canadian and swiss patients with phosphofructokinase deficiency. *American journal of human genetics* **1995**, *56*, 131-141.

4. Longtine, M.S.; McKenzie, A., 3rd; Demarini, D.J.; Shah, N.G.; Wach, A.; Brachat, A.; Philippsen, P.; Pringle, J.R., Additional modules for versatile and economical pcr-based gene deletion and modification in saccharomyces cerevisiae. *Yeast* **1998**, *14*, 953-961.
