# Supplementary material for: In Vitro and In Vivo Studies on the Structural Organization of Chs3 from Saccharomyces cerevisiae
Source: Int J Mol Sci. 2017 Mar 25;18(4):702. doi: 10.3390/ijms18040702 (PMC5412288; doi:10.3390/ijms18040702)
Supplement: Supplementary file 1 [file ijms-18-00702-s001.zip › Legends to Supplemental Figures.docx]

**SUPLLEMENTAL DATA**

***Figure legends***

**Supplemental Figure S1**. Topology predictions of Chs3 generated by various computer algorithms. The number of predicted TMHs varies between 5-8 TMHs. Also the orientation of soluble domains is unclear.

**Supplemental Figure S2.** ClustalΩ alignment of the C-terminal homologous region of Chs2 and Chs3 from *S. cerevisiae* (ScCHs2/3), NodC from *Rhizobium leguminosarum* (RlNodC), and BcsA from *R. sphaeroides* (RsBcsA). The conserved motifs of the catalytic site (Q(Q/R)XRW, EDR, SWG(D)TK) are highlighted in blue, the IF3 helix in green, and the finger helix in pink. Conserved TMHs (TM5 and TM6) forming part of the polymer conducting channel are colored cyan. Regions highlighted in red indicate two additional TMHs present in ScChs2. Asterisks indicate identical and dots more (:) or less (.) similar amino acids at the respective position.

Supplemental Figure S3. 3D structure predictions of chitin synthases generated by PHYRE2. As a template for structure predictions the crystal structure of the bacterial cellulose synthase BcsA was used. Note the highly conserved membrane-attached helix (purple) which was originally predicted as a TMH.

**Supplemental Figure S4.** CFW drop tests to test functionality of epitope-tagged Chs3 versions. CFW binds to chitin and inhibits the growth of the wildtype yeast, while *chs3*Δ strains grow properly. Serial dilutions of *chs3*Δ cells (with givers titers) expressing the indicated version of myc-tagged Chs3 were grown on SD^-Leu^ plates in the presence or absence of CFW for 24 h.

Supplemental Figure S5: Functional complementation of Venus-tagged Chs3 and BiFC negative controls. (A) Functional complementation of strains expressing Chs3^VN^ and /or Chs3^VC^. All strains were transformed either with the empty pRS415 vector or with pRS415 Chs3^VC^ and grown on selective SD^-Leu^ media. Yeasts were grown overnight in liquid SD^-Leu^ medium and diluted to a concentration of 10^7^ cells/ml. Two µl of the initial suspensions and serial 10-fold dilutions were dropped onto SD^-Leu^ plates with or without CFW (50 µg/ml). The growth of the strains was analyzed after 2 days of incubation at 30° C. (B) Fluorescence images of yeast cells expressing Chs3^VN^ or Chs3^VC^. Venus halves expressed individually show no fluorescence. Scale bars, 5 μm.

Supplemental Figure S6. Expression and functional complementation of truncated Chs3 versions and BiFC negative controls. (A) Fluorescence images of yeast cells expressing Chs3^VC^ truncations. Venus halves show no fluorescence when expressed alone. (B) Evidence of the expression of the shortened Chs3 versions by western blot using α-GFP. (C) Functional complementation of different truncated Chs3 proteins expressed in a *chs3*Δ strain. All strains were transformed either with the empty pRS415 vector or with pRS415 Chs3^VC^ truncations to grow on selective SD^-Leu^ media. Yeasts were grown overnight in liquid SD^-Leu^ medium and diluted to a concentration of 10^7^ cells/ml. Two µl of the initial suspensions and serial 10-fold dilutions were dropped onto SD^-Leu^ plates with or without CFW (50 µg/ml). The growth of the strains was analyzed after 2 days of incubation at 30° C.

**Supplemental Figure S7.** Serial dilution assays in the presence (-) or absence (+) of CFW. The assays were performed on SD^–Leu^ agar dishes plated without inhibitor (A) or with each 3 mg of the chitin synthesis inhibitors etoxazole (B), hexythiazox (C), clofentezine (D) and diflubenzuron (E). Similar results were obtained using 0.3 mg and 1 mg of the inhibitors.
