# Supplementary material for: In Vitro and In Vivo Studies on the Structural Organization of Chs3 from Saccharomyces cerevisiae
Source: Int J Mol Sci. 2017 Mar 25;18(4):702. doi: 10.3390/ijms18040702 (PMC5412288; doi:10.3390/ijms18040702)
Supplement: Supplementary file 1 [file ijms-18-00702-s001.zip › Supplemental Table 1.docx]

**Supplemental Table 1.** Strains

| **Strain** | **Genotype** | **Source or reference** |
| --- | --- | --- |
| BY4742 Δ*chs4* | MATα ; *his3*Δ*1*; *leu2*Δ*0*; *lys2*Δ*0*; *ura3*Δ*0* YBL061c::kanMX4 | Euroscarf [1] |
| BY4741 | MATa *his3*Δ*1* *leu2*Δ*0* *met15*Δ*0* *ura3*Δ*0* | Euroscarf [1] |
| BY4741 Δ*chs3* | MATa *his3*Δ*1* *leu2*Δ*0* *met15*Δ*0* *ura3*Δ*0* YBR023c::kanMX4 | Euroscarf [1] |
| BY4741 *CHS3^VN^* | MATa *his3*Δ*1* *leu2*Δ*0* *met15*Δ*0* *ura3*Δ*0* YBR023c-VN-hisMX6 | This study |
| BY4741 *CHS3^VN^* Δ*chs4* | MATa *his3*Δ*1* *leu2*Δ*0* *met15*Δ*0* *ura3*Δ*0* YBR023c-VN-hisMX6 YBL061c::KlURA3 | This study |
| BY4741 *CHS3^VN^* Δ*chs5* | MATa *his3*Δ*1* *leu2*Δ*0* *met15*Δ*0* *ura3*Δ*0* YBR023c-VN-hisMX6 YLR330w::KlURA3 | This study |
| BY4741 *CHS3^VN^* Δ*chs6* | MATa *his3*Δ*1* *leu2*Δ*0* *met15*Δ*0* *ura3*Δ*0* YBR023c-VN-hisMX6 YJL099w::KlURA3 | This study |
| BY4741 *CHS3^VN^* Δ*chs7* | MATa *his3*Δ*1* *leu2*Δ*0* *met15*Δ*0* *ura3*Δ*0* YBR023c-VN-hisMX6 YHR142w::KlURA3 | This study |
| BY4741 *CHS3^VN^* Δ*bni4* | MATa *his3*Δ*1* *leu2*Δ*0* *met15*Δ*0* *ura3*Δ*0* YBR023c-VN-hisMX6 YNL233w::KlURA3 | This study |
| BY4741 *CHS3^VN^* Δ*pfa4* | MATa *his3*Δ*1* *leu2*Δ*0* *met15*Δ*0* *ura3*Δ*0* YBR023c-VN-hisMX6 YOL003c::KlURA3 | This study |
| BY4742 *CHS3^VN^* Δ*chs4* | MATα ; *his3*Δ*1*; *leu2*Δ*0*; *lys2*Δ*0*; *ura3*Δ*0* YBR023c-VN-hisMX6 YBL061c::KlURA3 | This study |

1. Winzeler, E.A.; Shoemaker, D.D.; Astromoff, A.; Liang, H.; Anderson, K.; Andre, B.; Bangham, R.; Benito, R.; Boeke, J.D.; Bussey, H.*, et al.*, Functional characterization of the s. Cerevisiae genome by gene deletion and parallel analysis. *Science* **1999**, *285*, 901-906.
