# Supplementary material for: In Vitro and In Vivo Studies on the Structural Organization of Chs3 from Saccharomyces cerevisiae
Source: Int J Mol Sci. 2017 Mar 25;18(4):702. doi: 10.3390/ijms18040702 (PMC5412288; doi:10.3390/ijms18040702)
Supplement: Supplementary file 1 [file ijms-18-00702-s001.zip › Supplemental Table 2.docx]

**Supplemental Table 2.** Oligonucleotides

| **Name** | **Sequence (5’-3’)** | **Description** |
| --- | --- | --- |
| **BiFC** | | |
| SG132 | TTCTCAATCGGAAGGAGGAAAGTGACTCCTTCGTTGCAGCTGGTTCCGCCATGGTGAGCAAGGGCGAG | Chs3^VN^, chromosomal |
| SG146 | TCAACTTGTAAGTATCACAGTAAAAATATTTTCATACTGTTCATCGATGAATTCGAGCTCGTT |  |
| **ORF deletion** | | |
| SG176 | TTAGCTTGCATGTTACGTTTCCGTTTTAGAACCTGGTCGAGCCAGCTGAAGCTTCGTAC | *chs5* deletion |
| SG177 | TTCTTCTTATTCTTCTTATTCTTCTTCTTATTACTGTTGCTAGGCCACTAGTGGATCTGATATC |  |
| SG201 | CCTTTCTTAAGCTGTTGGTGCAAAAAAGGATTACATCTATGCCAGCTGAAGCTTCGTAC | *chs6* deletion |
| SG202 | GTACTGTGTGATCTAGGTCGGCTATCCACGCAAGCATGGCTAGGCCACTAGTGGATCTGATATC |  |
| NV1 | TCAAAAATTGTGGTTTTGTTAATACAAAAGCTGATATATAGTACGCTGCAGGTCGACAACC | *chs7* deletion |
| NV2 | ATTTCACATAAATCCATTATCATACACCACGTCACCGTCCATAGGCCACTAGTGGATC |  |
| SG172 | ATGTCGGATAGTATTTCAGATTCAAAGTCCTCAGAACTTTTGCCAGCTGAAGCTTCGTAC | *bni4* deletion |
| SG173 | CTAATAAAAATGTGTGTAACATCGCGAATCATCGTGTACTTTAGGCCACTAGTGGATCTGATATC |  |
| SG182 | TGATGGTGTATAACATGAATTTTAAAAACTGAGTTGATCGGCCAGCTGAAGCTTCGTAC | *pfa4* deletion |
| SG183 | TTCCATATCAACATCCACTCCAAAATCGTCTAGTGATTCATAGGCCACTAGTGGATCTGATATC |  |
| **Fluorescence organelle marker construction** | | |
| SG219 | TACTCACCCGGGTTAATTATGGTGAGCAAGGGCGAGGAG | *mCherry* amplification for pAG503 |
| SG220 | TACTCAGGCGCGCCCTACTTGTACAGCTCGTCCATG |  |
| SG209 | TACTCAGGTACCACTTCACTTAAATGTATATC | *sec66* amplification |
| SG210 | TACTCAGGATCCCATTGACTAATCTTCCATCAT |  |
| SG236 | TACTCACACTACGTGATCGCAATACTAATTTATTATAT | *chc1* amplification |
| SG237 | TACTCACCCGGGGATCCCAAATCCTGTGGGTTGAA |  |
| **Chs3 and VC amplification** | | |
| SG155 | TACTCAGGATCCCCGGGTTAATTAACGACAAGCAGAAGAAC | *VC* amplification |
| SG156 | TACTCACCATGGCTACTTGTACAGCTCGTCCATG |  |
| SG106 | TACTCAGGATCCGAATGACCGGCTTGAATG | *chs3* amplification |
| SG159 | TACTCAGGATCCATGTACATTGGTGCGATTG | *chs3* amplification |
| SG160 | TACTCACCCGGGATCTTTAACCATTTCAGC | *chs3* amplification |
| SG161 | TACTCACCCGGGTGCAACGAAGGAGTCAC | *chs3* amplification |
| **Chs3^13myc^ construction** | | |
| SG106 | TACTCAGGATCCGAATGACCGGCTTGAATG | *chs3* amplification |
| SG107 | TACTCACCGCGGTGCAACGAAGGAG |  |
| SG100 | TACTCACCGCGGCCCGGGTTAATTAACGGTG | *13myc* amplification |
| SG102 | TACTCAGAGCTCGCATGCGCTCTTCACTAGTGATTGATTAATTTTTG |  |
| **Chs3^3myc^ construction** | | |
| SG221 | P-CCCGGGAAGACAAATGGCGTGGAG | SmaI site insertion for Chs3^3myc(195/196)^ |
| SG222 | P-TCCTTCTTTGGCATCCCGC |  |
| SG223 | TACTCACCCGGGCAACGGTGAACAAAAGCTAATC | 3myc amplification for Chs3^3myc(195/196)^ |
| SG224 | TACTCACCCGGGACGGGCAAATCTTCTTCAG |  |
| SG238 | P-CCCGGGGTTGAAGTAGATTCAGACAC | SmaI and SacI site insertion for Chs3^3myc(263/264)^ |
| SG242 | P-GAGCTCGTCTTGTATACCGGAACGCG |  |
| SG232 | TACTCACCCGGGAACGGTGAACAAAAGCTA | 3myc amplification for Chs3^3myc(263/264)^ and Chs3^3myc(729/730)^ |
| SG244 | TACTCAGAGCTCAACGGTGAACAAAAGCTA |  |
| SG141 | TACTCATCTAGAGGGTTAATTAACGGTGAAC | 3myc amplification for Chs3^3myc(372/373)^ |
| SG142 | TACTCACCCGGGCAAATCTTCTTCAGAAATCAAC |  |
| SG143 | TACTCACCCGGGAACTTGATTGTTTATAATGGCGAC | Chs3 amplification for Chs3^3myc(372/373)^ |
| SG144 | TACTCAGCATGCCTATGCAACGAAGGAGTCAC |  |
| SG234 | P-TACTCACCCGGGTGGTCTAATAATATTCAAAC | SmaI site insertion for Chs3^3myc(503/504)^ |
| SG235 | P-TACTCACCCGGGATCCTCGATATCGTTTG |  |
| SG232 | TACTCACCCGGGAACGGTGAACAAAAGCTA | 3myc amplification for Chs3^3myc(503/504)^ and Chs3^3myc(1082/1083)^ |
| SG233 | TACTCACCCGGG GGGCAAATCTTCTTCAG |  |
| SG240 | P-CCCGGGGTGGCATCAGGCTCTAAAA | SmaI and SacI site insertion for Chs3^3myc(729/730)^ |
| SG243 | P-GAGCTCTGCCACATAGGAGTAAGG |  |
| SG215 | TACTCAGGTACCCAACGGTGAACAAAAGCTAATC | 3myc amplification for Chs3^3myc(922/923)^ |
| SG216 | TACTCAGGTACCACGGGCAAATCTTCTTCAG |  |
| SG225 | P-CCCGGGTGGTCGTACCTATGGTGGAT | SmaI site insertion for Chs3^3myc(1082/1083)^ |
| SG226 | P-TCTCGTAGCAGTTATAACAA |  |
