# Supplementary figures and images for: In Vitro and In Vivo Studies on the Structural Organization of Chs3 from Saccharomyces cerevisiae
Source: Int J Mol Sci. 2017 Mar 25;18(4):702. doi: 10.3390/ijms18040702 (PMC5412288; doi:10.3390/ijms18040702)

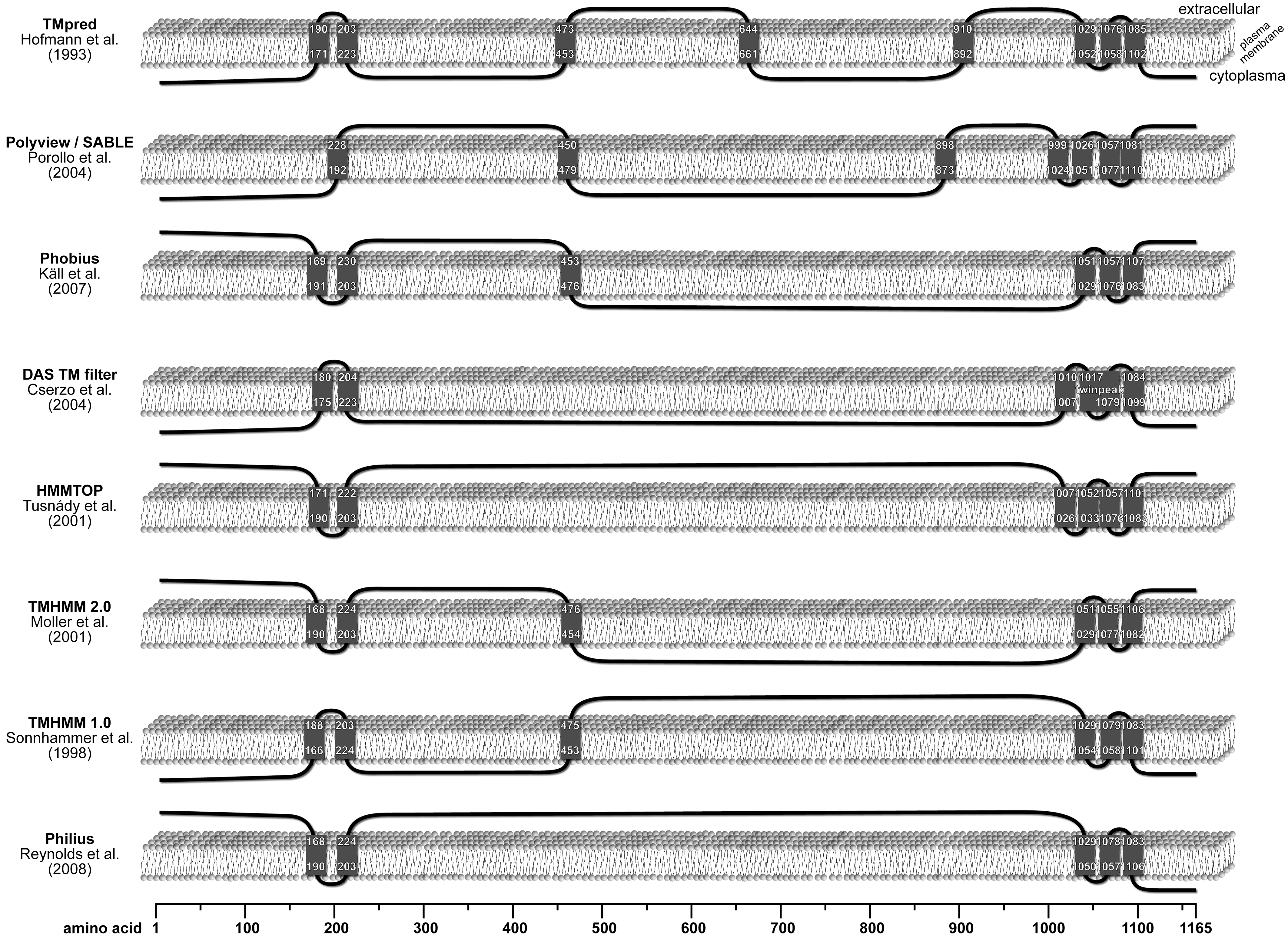

Supplement: Supplementary file 1 [file ijms-18-00702-s001.zip › Supplemental Figure S1.tif]

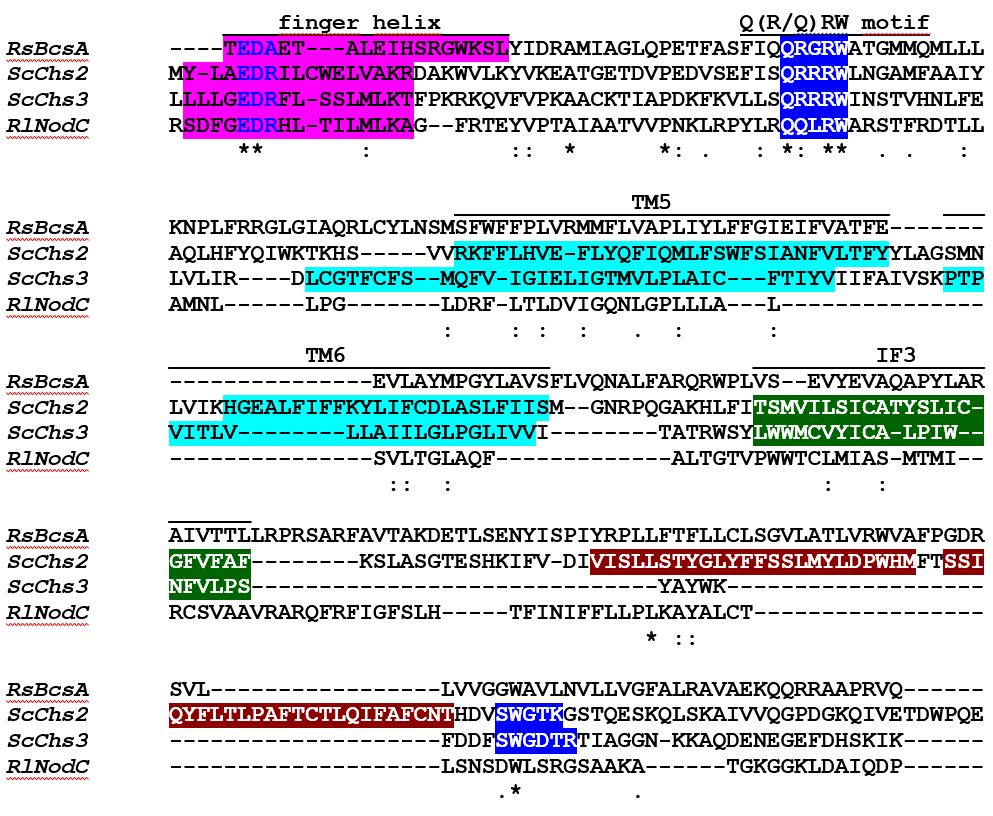

Supplement: Supplementary file 1 [file ijms-18-00702-s001.zip › Supplemental Figure S2.tif]

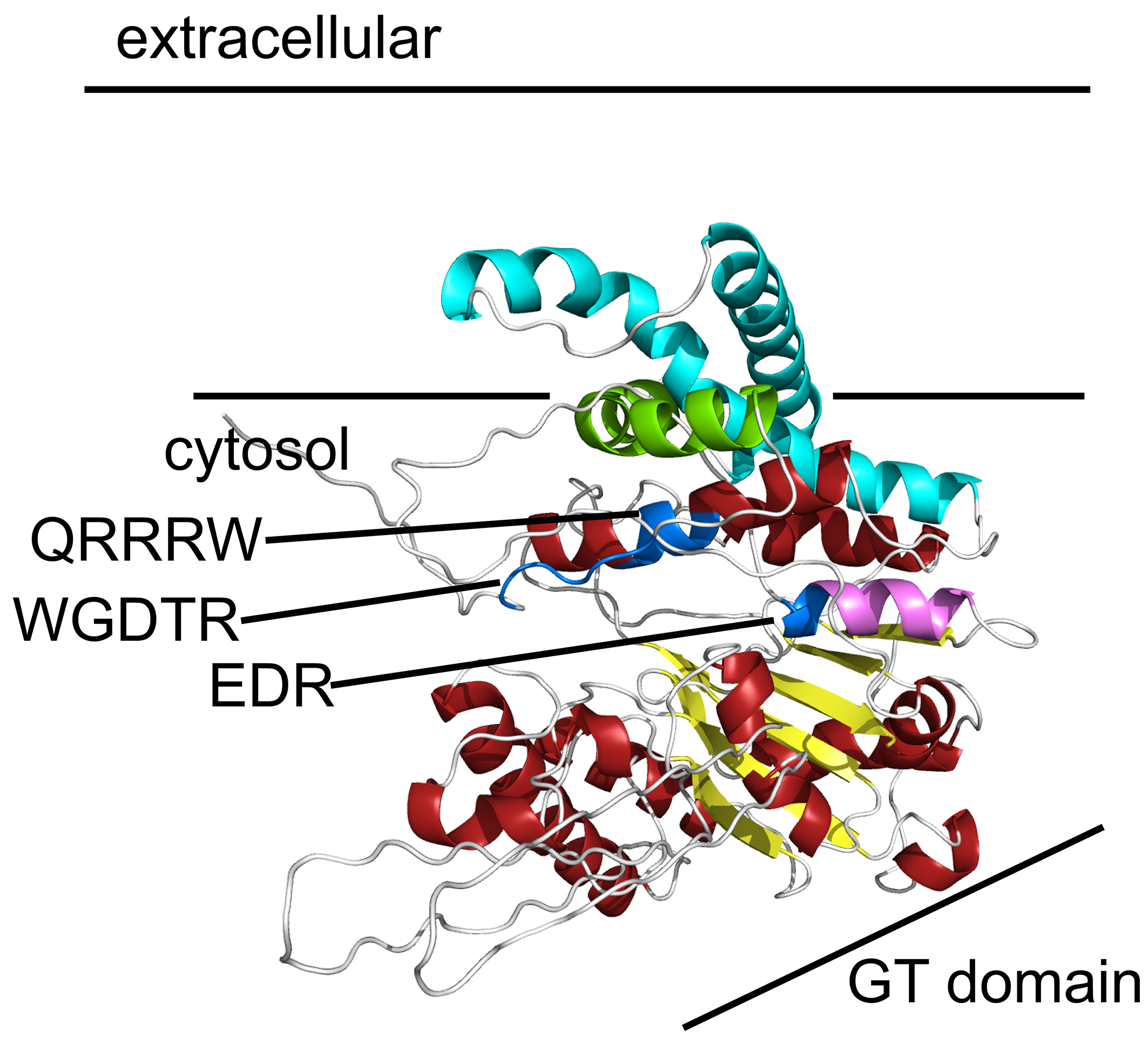

Supplement: Supplementary file 1 [file ijms-18-00702-s001.zip › Supplemental Figure S3.tif]

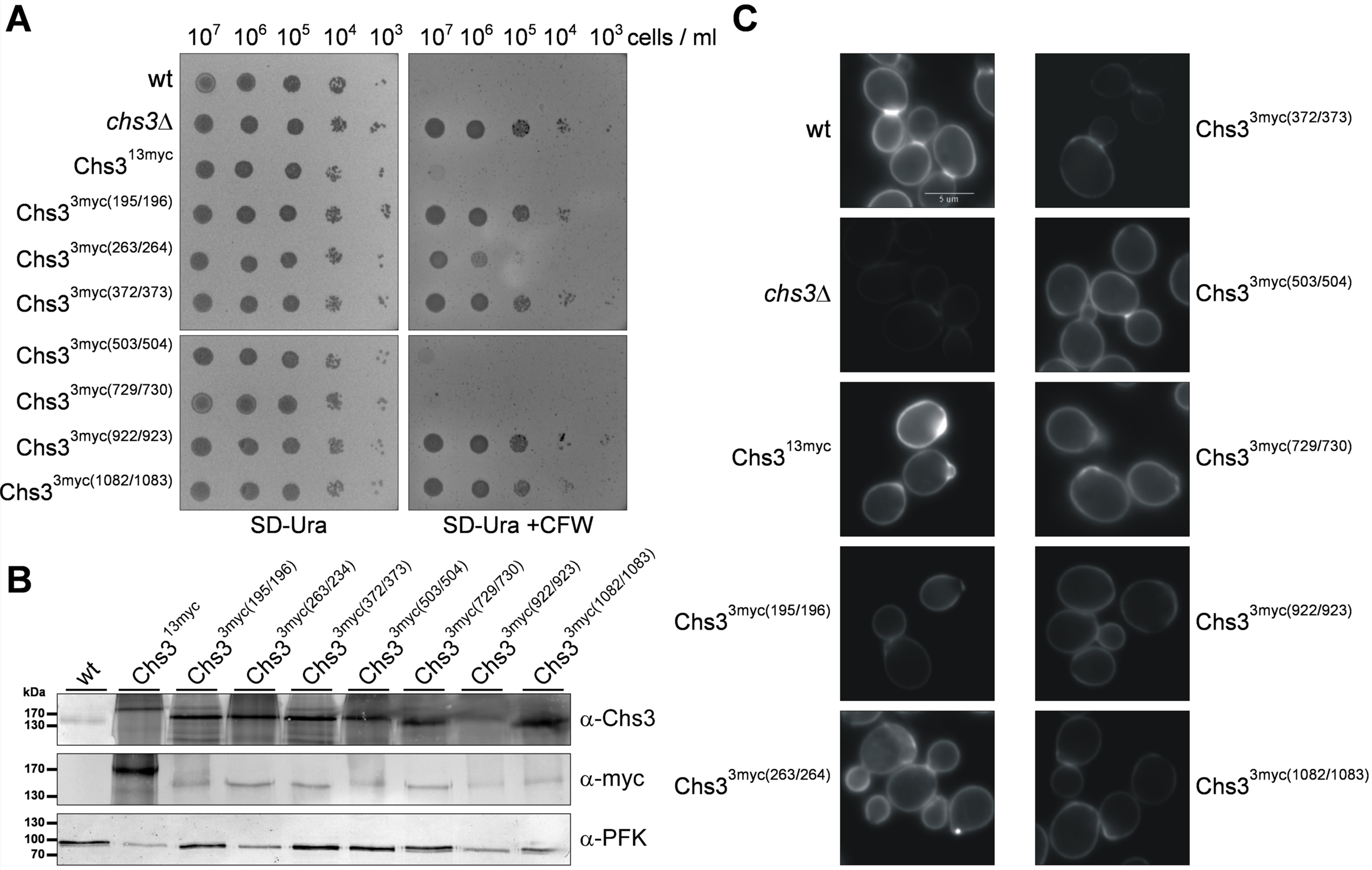

Supplement: Supplementary file 1 [file ijms-18-00702-s001.zip › Supplemental Figure S4.tif]

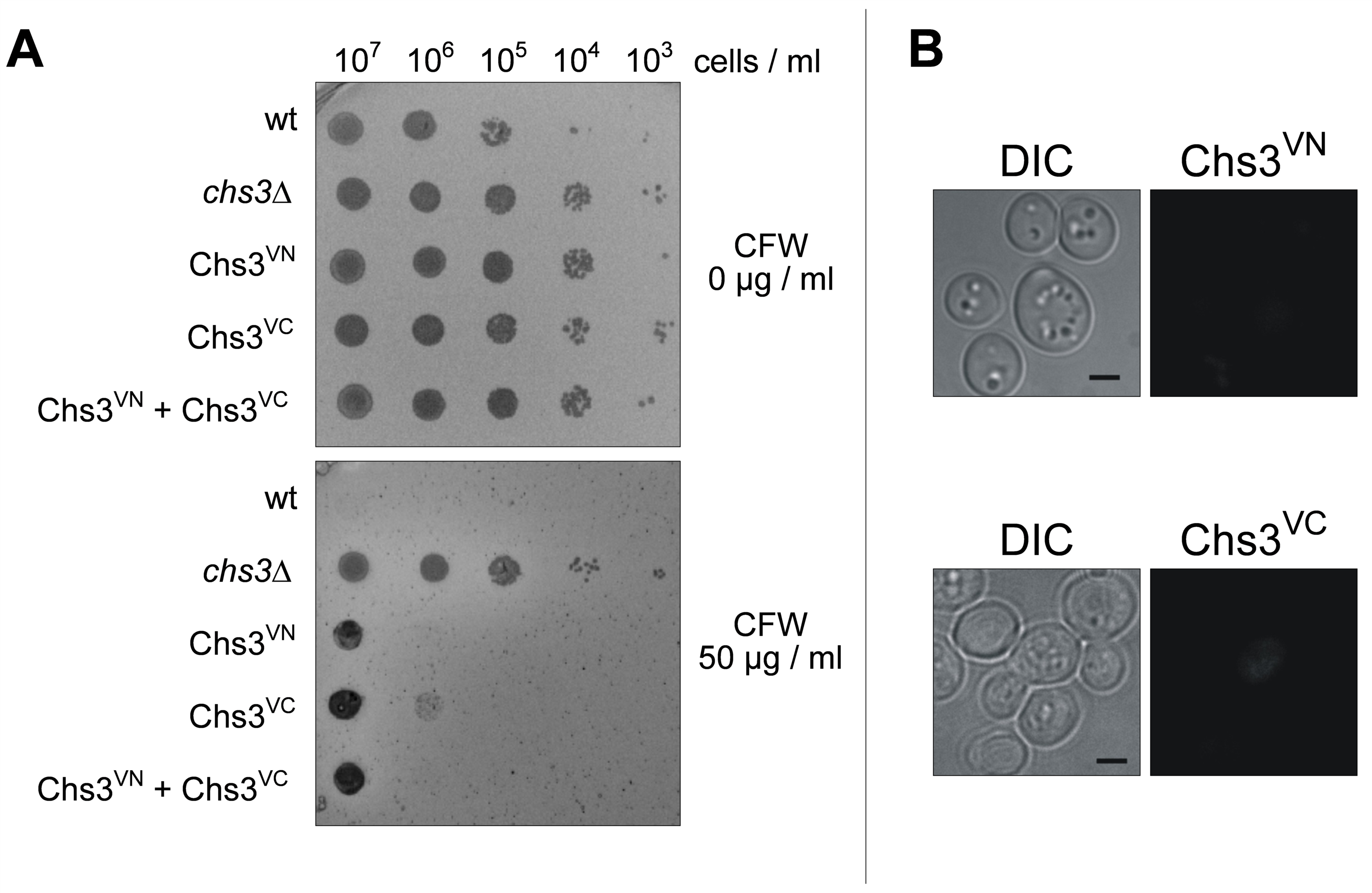

Supplement: Supplementary file 1 [file ijms-18-00702-s001.zip › Supplemental Figure S5.tif]

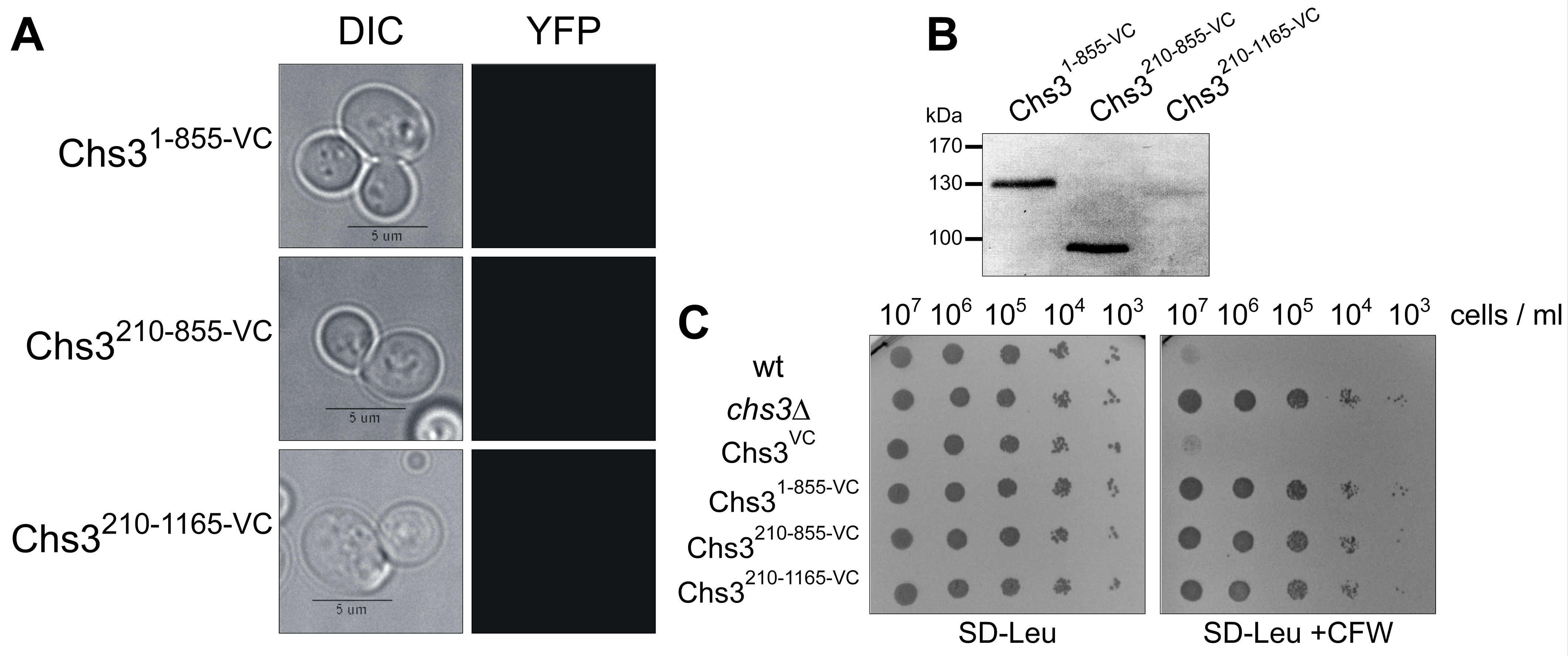

Supplement: Supplementary file 1 [file ijms-18-00702-s001.zip › Supplemental Figure S6.tif]

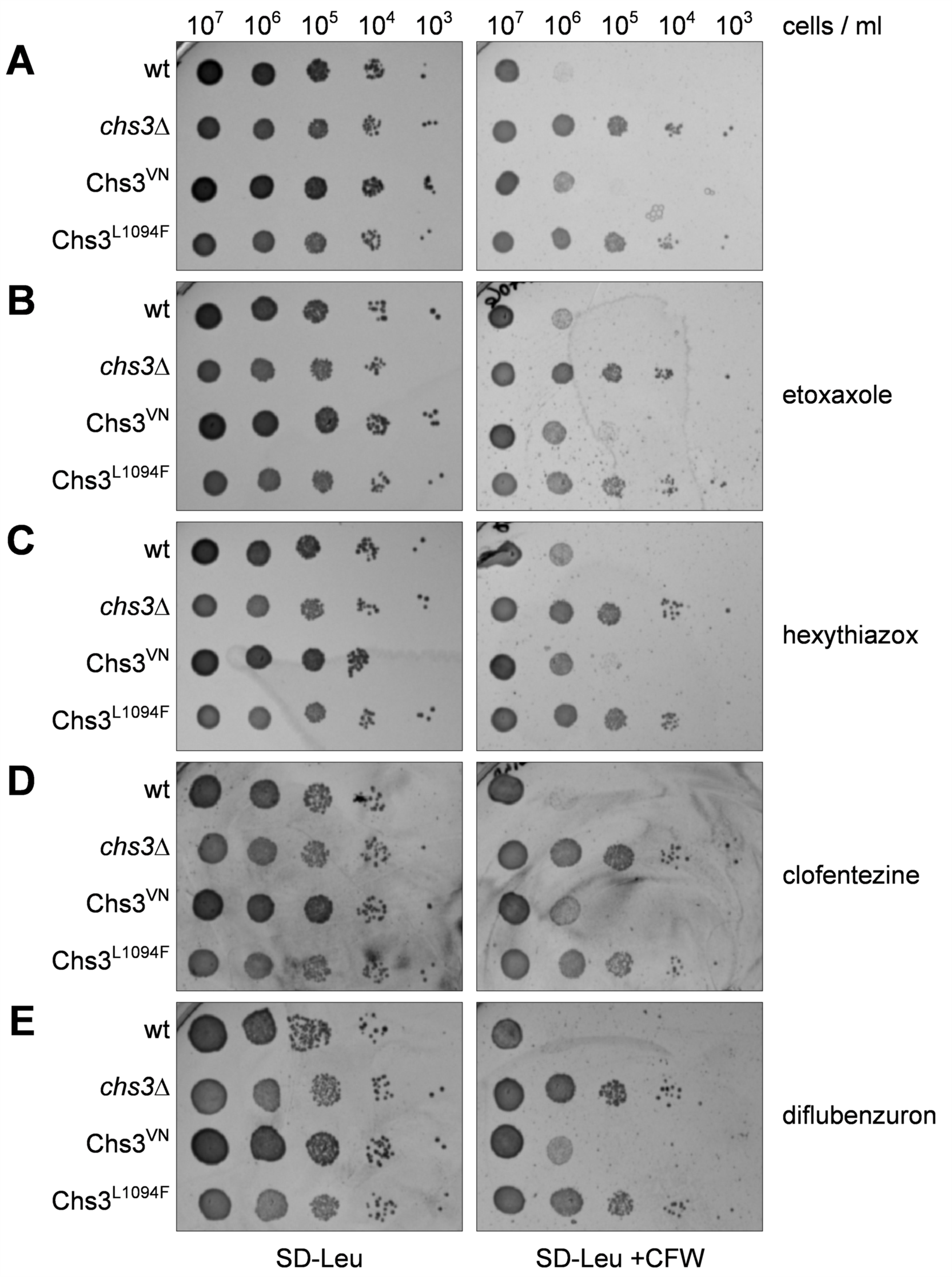

Supplement: Supplementary file 1 [file ijms-18-00702-s001.zip › Supplemental Figure S7.tif]
